# Flexible Strategies for Coping with Rainfall Variability: Seasonal Adjustments in Cropped Area in the Ganges Basin

**DOI:** 10.1371/journal.pone.0149397

**Published:** 2016-03-02

**Authors:** Christian Siderius, Hester Biemans, Paul E. V. van Walsum, Ekko C. van Ierland, Pavel Kabat, Petra J. G. J. Hellegers

**Affiliations:** 1 Alterra, Wageningen University and Research Centre, Wageningen, The Netherlands; 2 International Institute for Applied Systems Analysis, Laxenburg, Austria; 3 Environmental Economics and Natural Resources Group, Wageningen University, Wageningen, The Netherlands; 4 Water resources Management Group, Wageningen University, Wageningen, The Netherlands; Oklahoma State University, UNITED STATES

## Abstract

One of the main manifestations of climate change will be increased rainfall variability. How to deal with this in agriculture will be a major societal challenge. In this paper we explore flexibility in land use, through deliberate seasonal adjustments in cropped area, as a specific strategy for coping with rainfall variability. Such adjustments are not incorporated in hydro-meteorological crop models commonly used for food security analyses. Our paper contributes to the literature by making a comprehensive model assessment of inter-annual variability in crop production, including both variations in crop yield and cropped area. The Ganges basin is used as a case study. First, we assessed the contribution of cropped area variability to overall variability in rice and wheat production by applying hierarchical partitioning on time-series of agricultural statistics. We then introduced cropped area as an endogenous decision variable in a hydro-economic optimization model (WaterWise), coupled to a hydrology-vegetation model (LPJmL), and analyzed to what extent its performance in the estimation of inter-annual variability in crop production improved. From the statistics, we found that in the period 1999–2009 seasonal adjustment in cropped area can explain almost 50% of variability in wheat production and 40% of variability in rice production in the Indian part of the Ganges basin. Our improved model was well capable of mimicking existing variability at different spatial aggregation levels, especially for wheat. The value of flexibility, i.e. the foregone costs of choosing not to crop in years when water is scarce, was quantified at 4% of gross margin of wheat in the Indian part of the Ganges basin and as high as 34% of gross margin of wheat in the drought-prone state of Rajasthan. We argue that flexibility in land use is an important coping strategy to rainfall variability in water stressed regions.

## Introduction

South Asia’s climate is strongly influenced by land, ocean and atmosphere interconnections resulting in strong intra-seasonal [1–3], inter-annual [4,5] and decadal variability in rainfall [6,7]. The decadal cycle is now expected to approach a thirty-year dry epoch, with probability of below-average monsoon years increasing from once in every ten to fifteen years to once in every three years [8]. Climate change seems to reinforce this decadal drying: recent research linked cooling of the Tibetan anticyclone region and warming over the equatorial Indian Ocean during the recent decades to a weaker monsoon circulation [6]. Warming over the equatorial Indian Ocean might divert part of the monsoon rainfall to lower latitudes, away from the Indian subcontinent. Predictions for periods towards the end of the 21^st^ century are as yet inconclusive [9,10], with models generally suggesting an upward trend in regional rainfall but also an increase in inter-annual variability [11,12]. Whatever the long term trend, South Asia is facing a period with uncertainty in monsoon rainfall.

Food production in India, the largest country in South Asia, is highly dependent on the monsoon and inter-annual variability in monsoon rainfall. This is shown to cause large fluctuations in both monsoon-season crop production and production during the consecutive dry season [13–16]. Evaporative crop water demand is close to or even below mean annual rainfall in large parts of the region. Slight reductions in rainfall already lead to crop stress; when monsoon rainfall deficiency exceeds 10% compared to the long term average and consequently more than 20% of the country’s area is affected, the year is categorized as an all-India drought year [17]. However, at the local level sensitivity of food production to inter-annual rainfall variability can differ strongly. Whether a meteorological drought leads to an agricultural drought depends on local rainfall distribution and management practices for land and water like irrigation. Siderius et al. [18] showed that in the Ganges basin, the drier west is more affected than the wetter east, with the highly irrigated middle part of the Indo-Gangetic plain hardly showing any sensitivity.

Irrigation forms a buffer against rainfall variability, in both the Kharif (Monsoon/wet) and Rabi (winter/dry) season, and almost 30% of the cultivated area in India is now equipped for irrigation; more than half of this area is supported by groundwater, the rest by canal water and local reservoirs [19]. Presence of irrigation infrastructure alone, however, does not guarantee a continuous water supply from year to year. Large scale irrigation systems are not always effectively managed [20,21], with water often being over-allocated and supply insufficient for meeting total crop demand in the command area. Local storage facilities like shallow aquifers or village reservoirs (tanks), from which part of the irrigation water is drawn, are not always completely replenished during years with low rainfall [22]. As a result, in years of shortage a proportion of farmers will not have access to irrigation water and have to skip planting altogether. Others will have to choose: either they spread available water over a large area, facing a reduction in yield levels (risking total crop failure), or else they concentrate irrigation, maintaining high yield levels on a smaller area [18], and optionally supplementing income with rainfed crops [23,24]. Being flexible in leaving land fallow is a common coping strategy for dealing with water shortage. Between purely rainfed and fully irrigated agriculture there is a grey zone where cropped area, irrigated area and type of crops planted are dynamic variables depending on annual water availability and the cost of irrigation.

Such land and water use dynamics are usually not incorporated in hydro-meteorological and land surface models (e.g. [25–28]). Global and regional models used to assess the impact of water availability on food production typically focus on the impact of rainfall on yield, keeping the cropped area constant. Mostly, these models are calibrated and validated using long term average production values. Only recently [29] Kummu et al. analyzed the global impact of inter-annual rainfall variability on food production, indicating South Asia as one of the food security hot spots. In their study, as in many other studies, however, yields are simulated for a fixed land use pattern, without any inter-annual variation in cropped area or area irrigated. On those areas irrigated, optimum water supply is guaranteed, with water generally taken from an unlimited groundwater reservoir if surface water resources were insufficient (as e.g. in the dynamic hydrology-vegetation model LPJmL [27] or the Variable Infiltration Capacity (VIC) hydrologic model [28]). Only in some applications groundwater abstractions are restricted to a predefined volume [30], the size of which is hard to assess, however. Using this kind of optimal irrigation on a fixed land use pattern will probably lead to an underestimation of production variability and an overestimation of unsustainable groundwater use.

In this paper we explore the impact of flexible land use strategies for coping with rainfall variability. Flexibility in land use is in this paper defined as deliberate, seasonal adjustment of cropped area, by leaving land fallow or not. First, the contribution of cropped area variability to overall variability in rice and wheat production was assessed by applying hierarchical partitioning (ANOVA) on time-series of agricultural statistics (as explained in section 2.1). Cropped area was then introduced as an endogenous decision variable in a hydro-economic optimization model and subsequently we analyzed to what extent the model is capable of simulating the assessed inter-annual variability in cropped area and overall crop production, taking into account costs of irrigation and land use and the prices of crop produced (as explained in section 2.2). Finally, with the improved model, we quantified the value of flexibility, i.e. the foregone costs of choosing not to crop in years when rainfall is scarce. This value was assessed under current costs and price conditions, with and without cost of family labor. We focused on the Indian part of the Ganges basin, one of the world’s major food producing regions, and a region where groundwater depletion and seasonal water stress are major issues of concern.

## Methodology and Data

### Assessing the nature of crop production variability using agricultural statistics

We first determined how area and yield contribute to variability in production, using long-term time series on annual crop production, yield and cropped area for the whole of India and the Indian part of the Ganges basin, from the Department of Agricultural Statistics (http://apy.dacnet.nic.in/). To distinguish year-to-year variation from long-term trends, these time-series were de-trended using 3^rd^ order polynomial regression, which best describes the increase in production since the 1950s and the slow-down since the 1990s. De-trended cropped area and yield vary due to yearly management decisions and climatic variability. Annual crop production is the product of both. Logically, a linear regression that seeks to explain production as a function of area and yield has a predictive power of 100% (i.e., R^2^ = 1). However, possible correlations can exist between area and yields (e.g. anticipated high yields lead to an increase in cultivated area). In order to determine the relative importance of area and yield in explaining production, the method of ‘hierarchical partitioning’ [31,32] was used. The method was applied at the national level to India, to the Ganges basin and to all districts within the Ganges basin.

As an indicator of variability of production of different crops at different spatial scales, the Coefficient of Variation (*CV*, in the remaining text expressed as a percentage) was used,

$$CV\;=\frac{\sigma_{prod}}{\mu_{prod}}*100$$ (1)

where *μ*_*prod*_ is the mean production and σ_prod_ is the standard deviation of production. *CV* was calculated at district, state and basin level. A single value of variability for all districts (states) was obtained by aggregating *CV*s of the individual districts (states), applying weighted averaging on the basis of production. State-level production, aggregated from district-level production values of districts within the Ganges basin, does not represent the area of the state outside of the Ganges basin.

For the Ganges basin we could use district-level production statistics for 1999 till 2008, the most recent period for which consistent records are available from the Department of Agricultural statistics website of the Government of India (http://apy.dacnet.nic.in/). Data for all-India rice and wheat were retrieved from the same source, stretching back till 1950. For Rajasthan, additional data on wheat production came from the website of the Indian Directorate of Wheat Development (http://dwd.dacnet.nic.in/wheat_prod1/wheat-annx3.pdf). Data on rice production before 1999 came from the Indian Directorate of Rice Development (http://drdpat.bih.nic.in/).

### Modelling variability in crop production

#### The hydro-economic model WaterWise

While traditional climate-driven crop models are proven to be well capable of simulating average crop yields, i.e. productivity per hectare (e.g.[33]) they lack the capacity to vary the size of the area cropped based on available water resources. The hydro-economic model WaterWise (WW) can assess variability in crop yield as well as cropped area. WW optimizes the total gross margin (total yield-over-cost), choosing the optimal combination of land use and water management options, given available water resources:

$$\begin{array}{l} Y_{TOT}\;=Y_{LU}\;-C_{LWM}\\ \text{with}\\ \;Y_{LU}\;=\;\sum_{\text{z},\text{u},\text{y},\text{s}}\;(Prod_{\text{z},\text{u},\text{y},\text{s}}\;*\;P_{\text{y},\text{u}}\;-\;C_{LU,u}\;*\;Ac_{\text{z},\text{u},\text{y},\text{s}})\\ \;C_{LWM}=\sum_{\text{z},\text{u},\text{y},\text{s}}\;(C_{IRRIz,u}\;*\;Ac_{\text{z},\text{u},\text{y},\text{s}}) \end{array}$$ (2)

where *Y*_*TOT*_ represents total gross margin (in Indian Rupee (Rp) /yr), *Y*_*LU*_ the profit from land use (Rp/yr) based on production (*Prod*, in ton) multiplied by price of product (*P*, Rp/ton) minus non-water costs *(C*_*LU*_, Rp/ha) multiplied by the cropped area *(Ac*, in ha), in season *s* of year *y* per land use *u* in hydrotope *z*. *C*_*LWM*_ are the costs of local water-management measures for supporting land use, i.e., the variable costs of local irrigation measures (*C*_*IRRI*_, in Rp/ha), depending on the amount of water used, multiplied by the cropped area *(Ac*, in ha).

**Fig 1:**
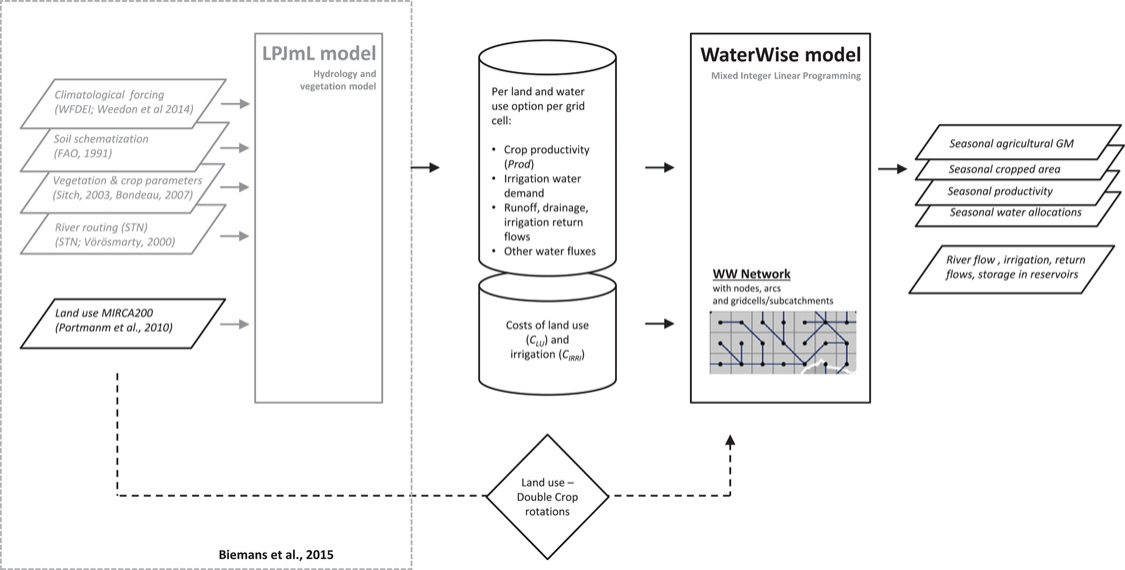
Model linkages between WaterWise and LPJmL. Climatological forcing is taken from WATCH-Forcing-Data-ERA-Interim (WFDEI). Schematization of soil, vegetation and river routing is based on global datasets. Land use is based on MIRCA. For the WaterWise model, MIRCA’s monthly land use was compiled into consistent double-crop rotations for WaterWise. Costs of land use per hectare and costs of irrigation per m^3^ of water used per ha are attached to crop productivity and water fluxes of each crop in each grid cell.

WW is a hybrid holistic model; production and water fluxes per ha of all land use and water management options are pre-processed by an off-line hydrology-vegetation model (here LPJmL [27,34]; see Fig 1 and next subsection). Land use is an endogenous variable in the WW model which allows for optimization of seasonal variability in land use. Unlike other hydro-economic models [35–37], WW does not explicitly contain a crop-water production function. In WW, the (sometimes extreme) nonlinearities between water and crop production are dealt with in the off-line column model. Crop productivity and water fluxes from the offline hydrology-vegetation model are then attached to continuous decision variables in WW that represent the area fraction for which a land and water management option is actually applied: associated with these variables are all the (time-dependent) water balance variables and crop production variables. By decreasing cropped area, production decreases, but also water demand is reduced and cultivation costs are avoided.

In this study we defined four land and water management options: *i*. leaving land fallow, resulting in no costs (only for Rabi season); *ii*. rainfed cultivation resulting in fixed costs of cultivation; *iii*. irrigation from surface water and; *iv*. irrigation from groundwater. Access to irrigation was derived from Portmann *et al*. [38], with the area with access to irrigation water corrected uniformly for each crop for the 10% increase in the decade since 2000, as based on the trend in the government statistics (GoI, 2012). Groundwater irrigation was constrained to a maximum of 66% of the irrigated area, based on FAO’s AQUASTAT data for the year 2001[39] (see also next subsection). The latter two options add additional costs depending on the amount of irrigation water supplied and the source of irrigation water. While WW does not contain a crop production function in the code itself, the combination of fallow, rainfed and irrigated crop production in the here used schematization implies, that WW has a choice between 3 discrete options along the crop-water production curve; zero production, ‘suboptimal’ rainfed production and optimum production at maximum water supply. As rainfall varies between the different 0.5° grid cells, at the aggregated level of a subbasin the model has, in effect, a whole range of options to choose from at different intervals along the crop-water production function, each with a different marginal return on water.

Because we were interested in present-day coping strategies, we blocked permanent land use conversion from one crop to the other in this study and instead focused solely on seasonal land and water management decisions. To realistically mimic only those seasonal land and water use decisions which are actually a farmers’ response to monsoon rainfall, the choice of leaving land fallow was restricted to the second cropping period, the so-called *Rabi* season. At the time of planting the *Rabi* crop, just after the monsoon, farmers usually have knowledge of available water resources. This in contrast to the first cropping period, the *Kharif*, when the monsoon has just started at the moment of planting and the availability of water resources over the growing season is still unknown [22,40]. As a result, monsoon rainfall totals could not be used as a decision-determining variable for the *Kharif* period. In this period, the model was only allowed to switch between irrigation and rainfed conditions. In the Ganges basin, about 60% of food crops are produced during *Rabi [34]*.

In terms of runoff routing and reservoir routines, WW is similar to other hydro-economic models like the Nile Economic Optimization Model [41], Ganges Economic Optimization Model [42], and Indus Basin model [37]. WW has been previously applied to the Nile Basin for quantifying the contribution of rainfed and irrigated agriculture to overall food security and, at a more local level, for solving complex issues of flood mitigation and water quality management in basins in Europe [43]. The WW model code is formulated within a Mixed Integer Linear Programming framework (MILP). The WW model equations have been implemented in Xpress-Mosel [44] and are summarized in S1 File. The complete formal description of the model, the model code and input and output data and documentation are available at www.waterwijs.nl and the DANS EASY public data repository (http://dx.doi.org/10.17026/dans-xea-j4wd).

#### Crop productivity, water fluxes and land use data

**Fig 2:**
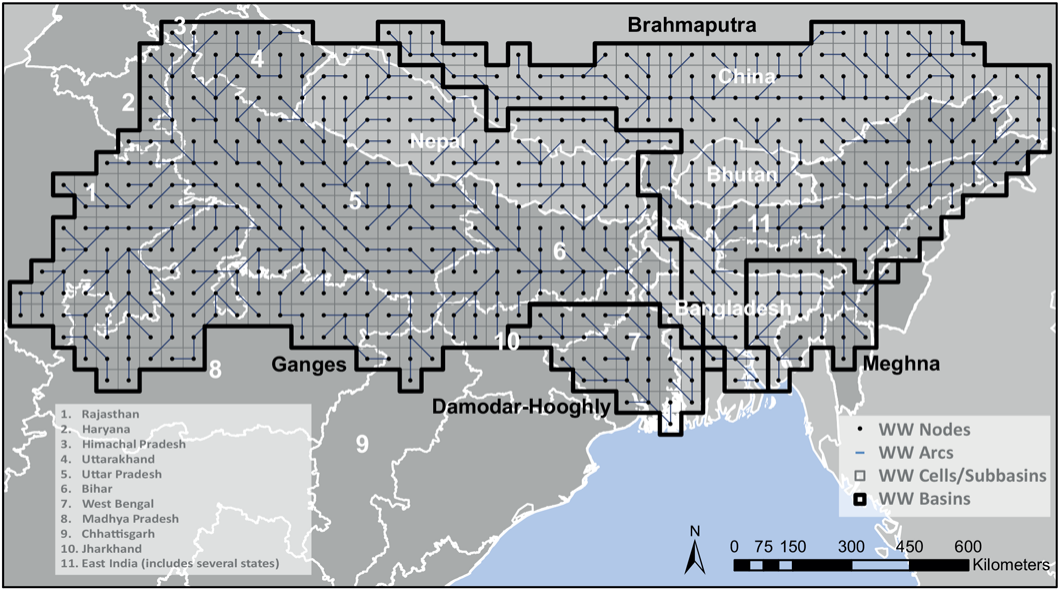
Model domain, with Indian states (dark grey) and other South Asian countries (light grey).

For the Ganges application, the hydrology-vegetation model LPJmL [27] was used as the off-line pre-processor of crop productivity and water fluxes at the grid cell level (0.5 degree resolution) (Fig 2). LPJmL has been widely applied in global and regional studies on water availability and food production [27,29,45–49]. LPJmL provided seasonal production, *Prod*_s_, per hectare for all crops and all four land and water management options in all gridcells of the LPJmL model for the Ganges domain (Fig 2). Associated with each combination was a daily irrigation water demand and all other day water fluxes; runoff, drainage and recoverable irrigation return flows to determine water availability and precipitation, evapotranspiration, soil moisture for both upper and lower soil compartments to complete the water balance for a check on consistency. We used the regional LPJmL model application described by Biemans et al. [34], which has seasonal crop productivity for the major food crops extensively calibrated and water demand validated at state level for both the *Kharif* and *Rabi* cropping seasons for the whole of South Asia. Climatological forcing in LPJmL for the 2000–2009 period was taken from the WFDEI meteorological forcing data set, a global meteorological dataset at 0.5° resolution [50].

WW was set-up for the Ganges-Brahmaputra-Meghna basin with a similar surface water and land surface grid structure as LPJmL, including the main reservoirs. The topological schematization of WW involves nodes *k*, arcs *j*, subbasins *r* and hydrotopes *z*, the latter representing agro-climatic zones with a certain soil type. In the Ganges application, subbasins are analogous to LPJmL gridcells (Fig 2), with hydrotopes analogous to the various crop classes in each LPJmL gridcell. While we set-up and ran the WW model for the whole Ganges-Brahmaputra-Meghna basin, our analysis focused on the Indian part of the Ganges basin for which consistent observed data are available. In the remainder of the article we will refer to this domain simply as ‘Ganges’. The grid cells of the used model are, at 0.5° resolution (~50km X 50km at the study site), similar in size to administrative districts, which warrants direct comparison of modelled *CV* based on grid cells, with observed *CV* based on district values. The model was run for a 10-year period, from 2000–2009, overlapping with the observed data.

The land use pattern was based on MIRCA2000 [38], which gives irrigated and rainfed cropped area for a total of 26 crops per month at a spatial resolution of 5-arc minutes. For the LPJmL South Asia application MIRCA’s monthly pattern was already aggregated to seasonal cropping patterns for *Kharif* and *Rabi* at 0.5 degree resolution [34]. To derive from these seasonal patterns the specific double-crop rotations in a gridcell, which are required in WW, we clustered *Kharif* rice, tropical cereals and maize with *Rabi* wheat, rice and pulses in each gridcell, according to the commonly used priority order in Table 1 (adapted from [51]). This lead to a total of 13 single crop rotation options (for *rabi* or *kharif*) and 5 double crop rotations in our model. Non-agricultural land use (nature, bare soil, rocks and glaciers, urban area) covers 59% of total area. In our analysis we focused primarily on the two staple crops–rice and wheat–in the Indian part of the Ganges basin. Rice is grown mostly during *Kharif*, whereas wheat is grown only during *Rabi* from November to April, being less resilient to high temperatures.

**Table 1:** Cropping pattern for the complete Ganges-Brahmaputra-Meghna basin, as adapted from MIRCA (Portman et al; [34]) and their priority order.

Crop id	Cropping period	Area (km^2^)	Area percentage		order
Rice_k_	Kharif	127043	7%		
Maize_k_	Kharif	27502	2%		
Pulses_k_	Kharif	25750	2%		
Oil crops_k_	Kharif	33105	2%		
Roots_k_	Kharif	60116	4%	16%	
Rice_r_	Rabi	13640	1%		
Maize_r_	Rabi	27502	2%		
Pulses_r_	Rabi	30596	2%		
Wheat_r_	Rabi	75674	4%		
Oil crops_r_	Rabi	41630	2%		
Roots_r_	Rabi	2077	0%	1%	
Rice_k_ Wheat_r_	Kharif—Rabi	103504	6%		1
Rice_k_ Rice_r_	Kharif—Rabi	40183	2%		2
Rice_k_ Pulses_r_	Kharif—Rabi	30596	2%		3
Tropical cereals_k_ Wheat_r_	Kharif—Rabi	15106	1%		4
Maize_k_ Wheat_r_	Kharif—Rabi	6317	0%	11%	5
Sugarcane	Whole year	23743	1%		
Other	Whole year	17824	1%		
Pasture	Whole year	4774	0%	3%	
Non agricultural land	Whole year	1005965	59%	59%	

Irrigation is only supplied in WW when total demand over the whole season can be realized. Irrigation water is taken from river flow, from groundwater and local runoff within the subbasin. In addition, cells within the main irrigation schemes of the basin can withdraw irrigation water from surface water not only from flow through the arc that directly crosses the cell but also from the main tributaries Yamuna, Upper Ganga and Ramganga, from which the large irrigation canals originate. Minimum flows to the Hooghly branch and to Bangladesh were inserted as minimum flow boundary conditions, each at 500 m^3^s^-1^.

#### Cost and farm-gate price data

Costs of cultivation and farm gate prices for the principal crops in India were derived from the Directorate of Economics and Statistics for the cropping year 2011/2012, the latest for which data was available (http://eands.dacnet.nic.in, last visited 31-10-2014). We did not intend to make a detailed full-scale analysis of India’s agro-economic performance, nor of the difference in profitability of agriculture between different states and therefore modified the data to single, simplified, crop-specific values for yields and prices for the whole basin (Table 2). In this way, crops competed for water, with differences in market conditions between states being neutralized. No distinction between *Kharif* and *Rabi* costs and prices was made.

**Table 2:** Costs and farm gate prices per hectare for 2011/12 and WW parameterization (Average costs and average prices from statistics represent the mean of all states, with minimum and maximum state-level values in between brackets; source http://eands.dacnet.nic.in).

	Costs (Rp ha^-1^)					Prices (Rp ton^-1^)			Break-even
	Statistics		WaterWise			Statistics		WaterWise	production
	Average Total costs		Land Use	Irrigation	Total costs	Average prices			(Ton ha^-1^)
Rice	19584	*(12544–29356)*	15000	5868	20868	10888	*(8970–13840)*	12500	1.8
Wheat	17498	*(11613–25538)*	15000	5008	20008	11666	*(10520–12790)*	12500	1.5
Tropical cereals	10467	*(6385–19081)*	7500	3532	11032	11992	*(8070–22000)*	12500	0.9
Pulses	10211	*(6678–13580)*	7500	3544	11044	35030	*(24870–61930)*	37500	0.3
Maize	11777	*(9192–16151)*	7500	4038	11538	9898	*(9100–10810)*	12500	1.2
Oil crops	11675	*(8618–15049)*	7500	6314	13814	24206	*(17460–33220)*	25000	0.5
Sugarcane	39274	*(31961–52947)*	30000	19215	49215	2205	*(2200–2100)*	2500	17.8
Other			30000	16543	46543			2500	

Cost and prices averages are not area-based. States included: Haryana, UP, Rajasthan, Uttarakhand, Bihar, Madhya Pradesh, West Bengal and Assam). Irrigation costs are based on maximum irrigation requirement (as calculated by LPJmL) times irrigation costs of 0.01 USD (which is about 0.6 Rupees at autumn 2014 exchange rates)

Costs are comprised of land use costs (*C*_*LU*_) per hectare and variable irrigation costs (C_*IRRI*_) based on the m^3^ of water used per ha. *C*_*LU*_ includes all actual expenses in cash and kind like fertilizer costs, irrigation charges and value of machinery, but excludes rental value of the land and value of family labor (A2 class, [52]). *C*_*IRRI*_ accounts for irrigation charges and hired machinery, diesel and electricity costs needed for irrigation. Applying a generally used value of 1 USD cent per m^3^ (~0.6 Rp) multiplied by the maximum amount of irrigation water applied as calculated by LPJmL gave maximum C_*IRRI*_ ranging from 3500 Rp per ha for pulses and tropical cereals to almost 20000 Rp for sugarcane. Cost of irrigation for sugarcane is this high as it also requires water in the hottest and driest months of the year. The ratio between *C*_*LU*_ and maximum C_*IRRI*_ is approximately 3 to 1 for rice and wheat. The same ratio was found in state-level statistics for states in the Ganges basin with high irrigation water use (i.e. Haryana, Uttar Pradesh).

Prices at farm gate level of rice (paddy), wheat, tropical cereals and several others crops vary around 12500 Rp ton^-1^. Oil crop prices are on average double and the price of pulses almost triple that amount. Yields in ton/ha are on average considerably lower for these crops, though, reducing their comparative advantage. Sugarcane prices are only a fifth of those for staple crops, but yield in ton per hectare for the raw product is a factor 10 to 20 higher. Due to its long growth period (12 months in the model, in reality sometimes longer) its water demand is high, though, and a stable water supply is required for a successful yield.

#### Scenarios

As a baseline, we ran WW in **simulation mode**, mimicking the production of rice and wheat as simulated by the LPJmL model (‘*WW-baseline’ variant*). In simulation mode, variation in area cropped is not allowed (*Ac*_z,u_ *=* constant) and groundwater resources are unlimited. Production is described as:

$$\begin{array}{l} {Prod}_{\text{z},\text{u},\text{y},\text{s}}\;=\;{yld}_{\text{z},\text{u},\text{y},\text{s}}\;*\;{Ac}_{\text{z},\text{u}}\;\text{and}\\ {yld\;}_{\text{z},\text{u},\text{y},\text{s}}\;=\;f_{1}(Crop,Soil,T,Rad,RH,Prec,Ir) \end{array}$$ (3)

where *yld*_z,u,y,s_ is seasonal crop yield (in ton per ha). Seasonal crop yield is influenced by the crop type *(Crop)*, soil conditions (*Soil*) and the meteorological variables temperature (*T*), incoming solar radiation (*Rad*), relative humidity (*RH*), precipitation (*Prec*) and access to irrigation (*Ir*); the latter is a binary condition and based on the MIRCA land use database, which indicates how much of the area for each crop is equipped for irrigation [38,53]. If equipped for irrigation, water demand is met either from surface water or from groundwater.

To explicitly allow for adjustment of cropped area in our model, we switched to running the model in **optimization mode**, including seasonal costs of land and water use and benefits of crop production. The seasonal decision to crop (or leave land fallow without any costs involved) or to irrigate then becomes an economic decision, influenced by costs of land and water use and economic yield of production *(‘WW-flexible’ variant*). Cropped area is now calculated as:

$${Ac}_{\text{z},\text{u},\text{y},\text{s}}\;=\;f_{2}({yld}_{\text{z},\text{u},\text{y},\text{s}},P_{\text{y},\text{u}},C_{LU,u},C_{IRRI},{qsupply}_{\text{z},\text{u},\text{y},\text{s}})$$ (4)

with *P*_y,u_ the price per ton yield (in Indian Rupee (Rp)/ton), *C*_*LU*,*u*_ is the cost of cultivation (in Rp/ha) and *C*_*IRRI*_ the cost of irrigation (in Rp/ha, depending on the m^3^ of irrigation water required per ha) and *qsupply*_,*z*,*y*,*s*_ is the available supply of irrigation water. Gross margin, i.e., production multiplied by prices minus costs, is optimized for the basin as a whole. This means, if given the flexibility to leave land fallow, an area is only cropped when benefits per ha exceed costs per ha, and when also, basin-wide, water cannot be used more productively elsewhere.

Finally, to further constrain the model and better mimic variability as observed, we restricted access to unlimited groundwater reserves by replacing part of it with virtual local storage reservoirs (VLSRs), representing shallow groundwater aquifers and storage in local reservoirs (ponds, village tanks) that are seasonally recharged by local runoff. Water availability in areas depending on VLSRs will fluctuate from year to year, limiting crop production in dry years, so that cropped area and production variability will increase. The decision to crop or to irrigate then becomes an economic decision influenced by seasonal water scarcity (*‘WW flexible-limited’ variant*).

The exact size and number of all open wells, ponds, tanks and water harvesting reservoirs and area that is irrigated by them is unknown. Groundwater irrigation in South Asia is largely unregulated with only limited government control and monitoring [54]. In a sensitivity analysis, we varied the volume of these VLSRs in combination with the area of cropland irrigated by them. The separate types of storage facilities are lumped in the model per subbasin. Volumetric capacity of a VLSR was calculated as the assumed depth of the reservoir multiplied by the area of cropland in a subbasin depending on it. For the depth of reservoirs we used a range from 0.01m to 1m. The area irrigated from VLSRs ranged from 0% to 66%, with area irrigated from deep groundwater reduced accordingly to maintain a total area irrigated from groundwater and VLSRs of 66%. We then compared the resulting range of production variability with observations and selected the parameter combination for which simulated variability approached observed variability.

## Results

### Variability in production of rice and wheat

**Fig 3:**
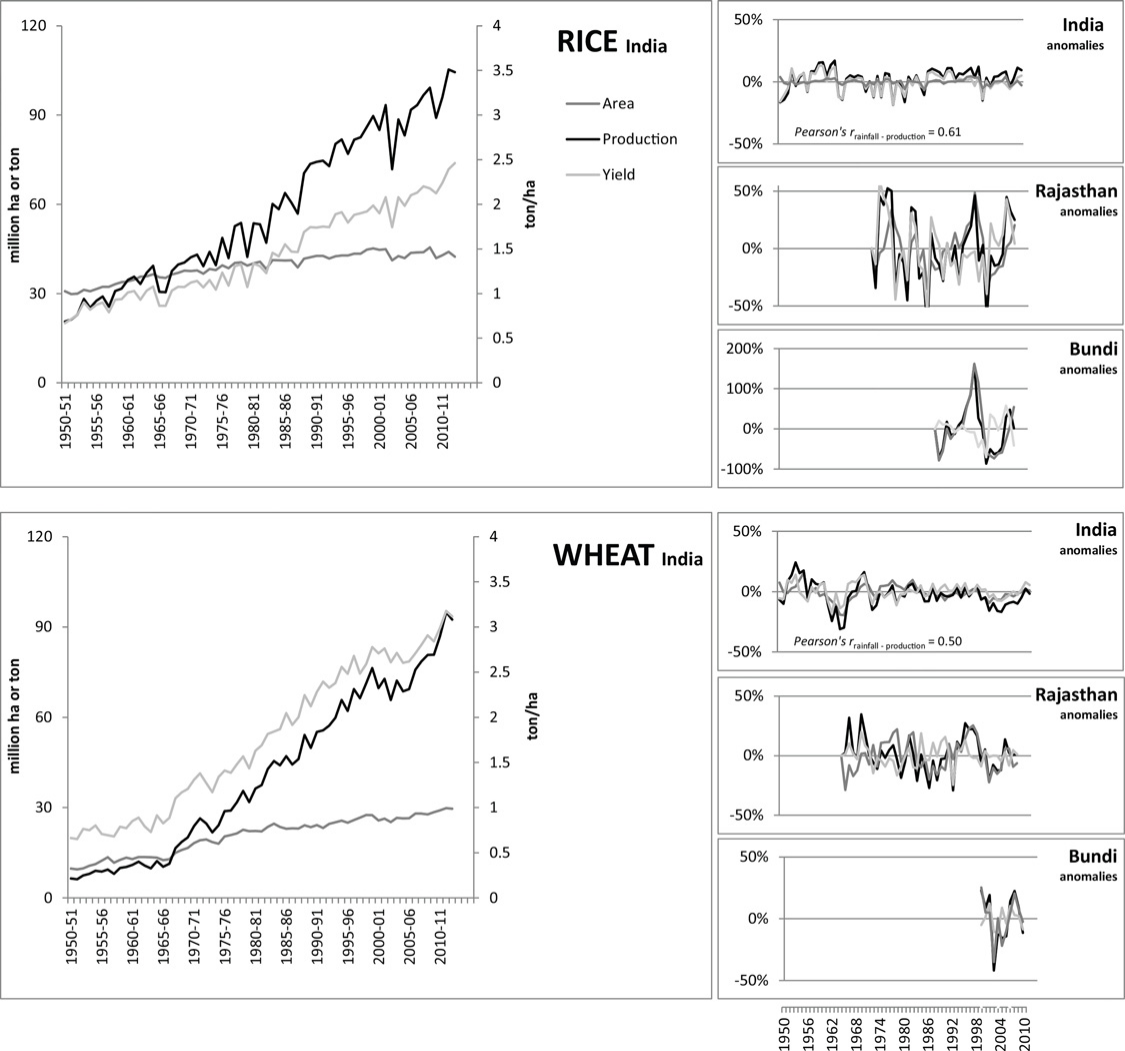
Crop production, yield and area for rice and wheat for India (left), and anomalies for India, state (Rajasthan) and district level (Bundi district in Rajasthan) (right) (data source GoI, 2012).

Trend-corrected cropped area, yield and production data for rice and wheat are shown for India in Fig 3. Both yield and area have increased over the past decades. While area has increased rather linearly, yields have increased more rapidly since the mid-sixties as a result of new high-yielding varieties and improved irrigation supply and nutrient inputs of the green revolution. From the 1980s the trend has continued mainly due to additional groundwater exploitation [55]. As a result of these increases, production has risen fourfold for rice and fifteen-fold for wheat; India has thus become self-sufficient in both commodities despite its rapid population growth. While the trend in yield and production increases seems to slow down since the end of the 1990s, favorable weather conditions still led to bumper crop yields in recent years.

After de-trending, the yearly anomalies in crop production, yield and area remain. Anomalies at all India level are presented in Fig 3, which also contains examples for the drought-prone state of Rajasthan and separately for its Bundi district, an important rice and wheat producing area. Clearly, variability in all three variables increases when going to a lower level of scale for both rice and wheat production. This is to be expected as variations in districts average out at state level, and variation between states average out when totalized at all-India level. For instance, annual rice production in a drought prone state like Rajasthan is influenced differently by rainfall anomalies than rice production in a cyclone prone state of West Bengal. Overall, at all-India level, fluctuations have increased over time in absolute terms, but decreased in relative terms. This is a result of the large increase in area, yield and production over the past decades (S2 File).

The relative contribution of cropped area fluctuations to overall production variability, as determined by the hierarchical partitioning method, also seems to increase when moving to the more local scale (Table 3). At all-India level, production anomalies are caused mainly by yield fluctuations and only partly by a fluctuating cropped area. Zooming in on Rajasthan and on Bundi the influence of area fluctuations increases. The same pattern can be seen when analyzing all districts in the Ganges basin over the period 1999–2009 and comparing district-level variability against basin variability. Overall, these figures show that cropped area adjustments are almost as important as fluctuations in yield in explaining production variability.

**Table 3:** Relative importance of cropped area and yield in explaining variability in production.

	rice			wheat		
	Area	Yield	time period	Area	Yield	time period
India	31%	69%	1950–2012	44%	56%	1950–2012
Rajasthan	39%	61%	1974–2009	69%	31%	1966–2009
Bundi district	92%	8%	1990–2009	74%	26%	1999–2009
Ganges basin total	39%	61%	1999–2009	34%	66%	1999–2009
Ganges district average*	51%	49%	1999–2009	43%	57%	1999–2009

* for all districts in the Indian part of the Ganges basin

### Modelling variability

#### A matter of costs and benefits

**Fig 4:**
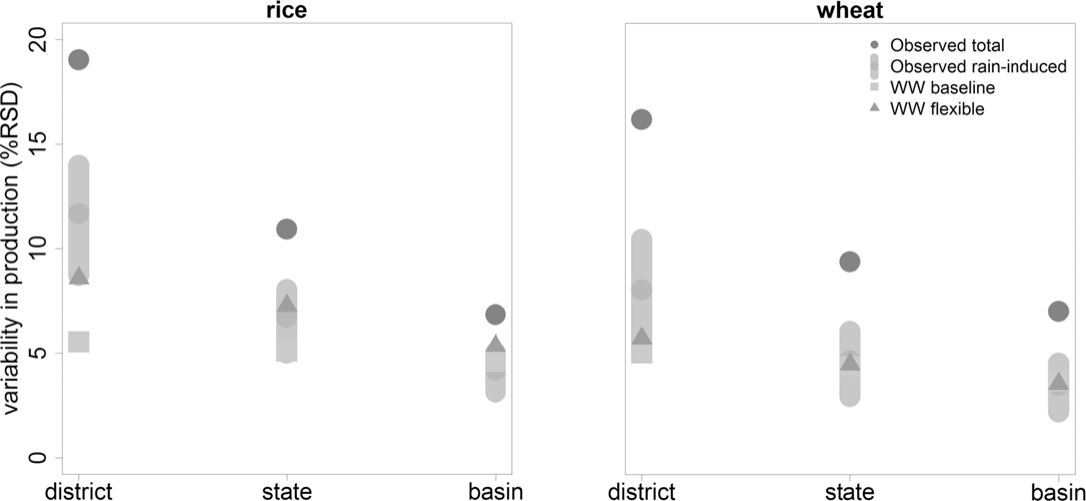
Variability in production (CV), averaged for district and states in the Ganges basin and the total basin, with observed total variability (‘Observed total,’ source MOA, 2012), variability correlated to rainfall (‘Observed rain-induced’, expressed as a range) and variability as simulated by WW without costs (“WW baseline” variant) and with costs and flexible land and water use (“WW flexible” variant).

Variability in crop production simulated by a hydro-meteorology-driven model should fall within the bandwidth of observed variability caused by rainfall (see S3 File for how this bandwidth was determined). With WW in simulation mode, using the exogenous land use from the LPJmL model and no costs attached to land or water use (the “*WW baseline*” variant), variability in production is clearly underestimated (Fig 4). As water resources are unlimited in this variant, production is optimal for all irrigated crops, resulting in a stable and overall very uniform production from year to year. The *CV* mainly reflects variability in rainfed yields or minor fluctuations in yield from irrigated areas due to fluctuations in agro-climatic parameters other than rainfall. The decreasing trend in variability in observations from district to basin level is hardly resembled.

Making seasonal land and water use an economic decision based on costs and benefits in WW, and allowing the model to choose the amount of land under cultivation in the *Rabi* season (“*WW flexible*” variant, Fig 4), improves simulated inter-annual variability in production considerably for rice, but hardly for wheat. A stronger increase in variability for rice is to be expected; yields per ha are on average lower than for wheat, especially in poorer states like Bihar, while costs of cultivation are in the same order and the amount of irrigation water required is often higher. Adding costs to land and water use and giving (the model) the opportunity to restrain from irrigation or planting a second crop when rainfall is scarce, thus, mainly affects rice production in states with low productivity. For wheat, which is more than 90% irrigated, the benefit of irrigation far exceeds the costs of irrigation. With sufficient irrigation water available, either from surface water or groundwater, the value of water will not be a limiting factor for wheat production under current price conditions.

#### A matter of groundwater access

**Fig 5:**
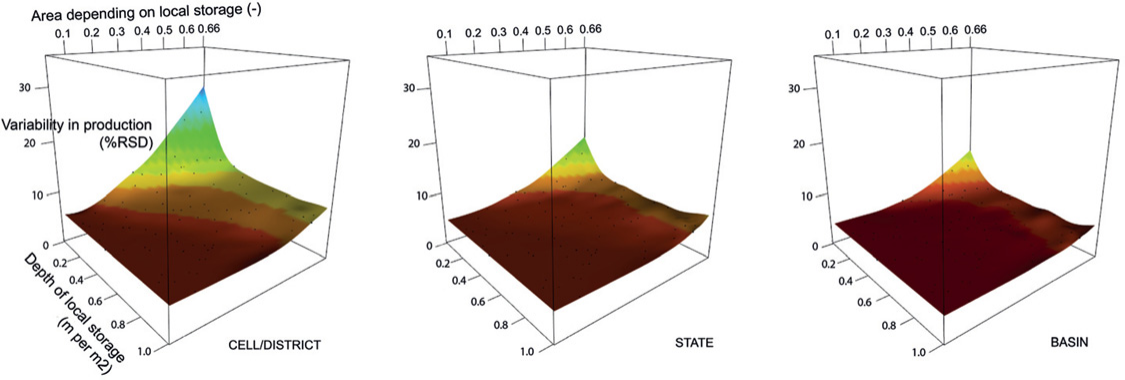
Variability in wheat production (CV) as function of size of virtual local storage reservoirs (expressed in m per m2 of area irrigated from the reservoirs) and area depending on them (as a fraction of total irrigated area, with the area with access to deep groundwater lowered accordingly to maintain a constant area irrigated from deep groundwater and virtual local storage reservoirs), at district/cell, state and basin level. Labels of axis at state and basin level are identical to those of cell/district level.

In order to increase simulated variability in wheat production, limiting access to water, rather than introducing a price to water use seems a necessary option to explore. Fig 5 shows variability in wheat production as a function of the volumetric capacity of local storage reservoirs (VLSR) and the dependency of wheat production on this local storage (rather than on unlimited ground water). At district level, variability increases to up to 30% *(CV*), when the volumetric local storage capacity approaches zero and deep groundwater irrigation is absent. In this extreme case, only surface water irrigation on the remaining 34% of irrigated area is available. District level results also show that even when there is a large VLSR capacity, deep groundwater is indispensable for buffering rainfall variability, as without it variability will not get below 10%. In regions with high cropping intensity and/or low rainfall, additional runoff to be stored in the local storage reservoirs is simply insufficient for providing enough water for all crops. With maximum access to groundwater, a constant production can be maintained and any remaining variability is caused mainly by variability of the small area under rainfed production and from climatic parameters other than irrigation water supply, like temperature. Variability at state and basin level show similar patterns of variability in production, but at a lower level.

**Fig 6:**
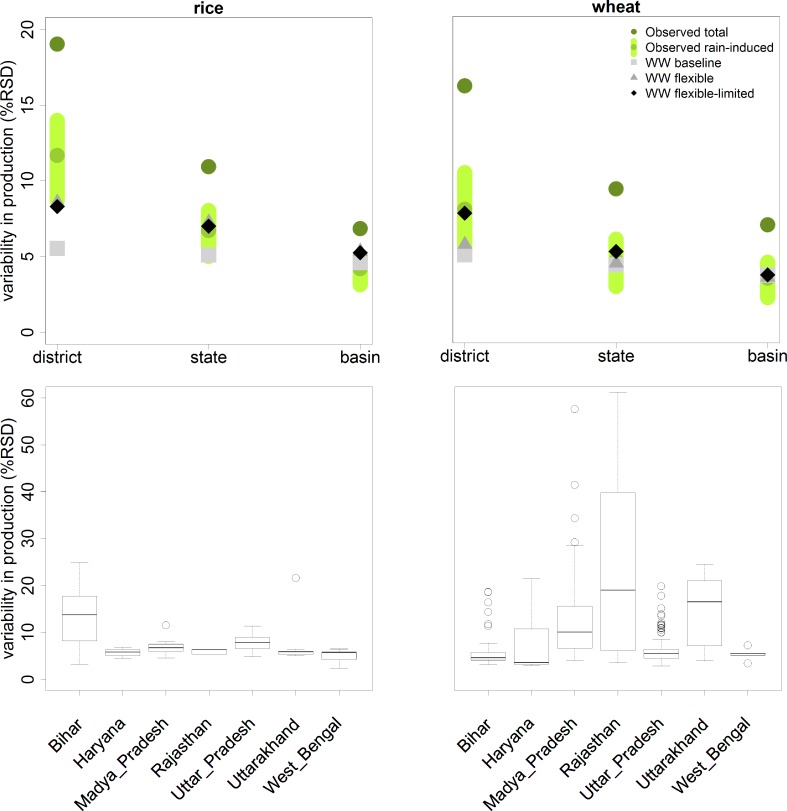
(A) Variability in production with limited deep groundwater(top) and (B) box-whisker plots of simulated variability in production (CV for the “WW flexible-limited” model variant) for the various cells within each state within the Ganges basin (bottom).

To improve our model, parameter values for VLSR depth and area irrigated by them should be chosen such that district variability for wheat approaches the expected *CV* of ~9%. With two parameters there is, however, a whole range of combinations possible that approach this variability in production (the yellow gradient in Fig 5 –district level). As a best guess estimate, we assumed that the area having continuous access to VLSR will be half the stated area from statistics (so 33% of the total irrigated area, with deep groundwater serving the other 33%). Local storage on this area should then be 150 mm to match observed variability in wheat at the district level. Simulated *CV* approaches, as Fig 6A shows, the mid of the range of expected variability in wheat production as caused by rainfall for all levels in this “*WW flexible-limited*” model variant. Especially at district level the simulation of variability improves considerably. Rice production is hardly affected by any combination of these parameters and simulated variability remains, at district level, at the lower end of the expected observed variability.

In Fig 6B variability for all individual cells, clustered per state, is shown. For rice, the average variability is rather constant over all states except for Bihar, a downstream state with a relatively low productivity. For wheat, the most extreme variability is found in the two more drought-prone states Rajasthan and Madhya Pradesh in the south-western part of the Ganges basin. Variability in wheat production in Uttarakhand is high as well, mainly because of a high percentage of rained wheat production in this mountainous state.

While simulated variability improved by introducing flexibility in cropped area, average production of rice and wheat was hardly affected (Table 4). Total rice yields in the Indian Ganges basin were reduced by less than 4%, while wheat production was reduced by just over 6%, despite introducing constraints on deep groundwater availability during the wheat producing season. While introducing more inter-annual variability, average simulated production, thus, remains close to official estimates with ~60% of Indian wheat and ~26% of rice produced in the Ganges basin. Overall, results in Table 4 show that variability in rice production can largely be explained by yield fluctuations, while variability in wheat production is a result of both area and yield fluctuations, which depends on the location in the basin. In upstream rainy Uttarakhand, variability in production is mainly due to fluctuations in yield, while in dry Rajasthan, with its high reliance on irrigation, fluctuations in area start to dominate once the model is given the freedom to vary it. Variability in yield decreases between the scenarios when area is allowed to vary; the model prefers to maintain high production per ha and to reduce costs by decreasing the amount of hectares during periods of shortage. This behavior appears to match reported coping strategies of farmers [22–24].

**Table 4:** Impact of model improvements (“*WW baseline*”, “*WW flexible*”, “*WW flexible-limited*”) on average rice and wheat production, cropped area, yield and gross margin per hectare, and their variability *(CV*), at state level and basin totals–Ganges basin domain only.

**rice**												
	**production (million tons)**			**area (million ha)**			**yield (ton/ha)**			**gross margin (Rp / ha)**		
**AVERAGE**	**baseline**	**flexible**	**flex limited**	**baseline**	**flexible**	**flex limited**	**baseline**	**flexible**	**flex limited**	**baseline**	**flexible**	**flex limited**
Bihar	6.4	5.9	5.7	4.7	4.3	4.2	1.4	1.4	1.3	-	32	30
Haryana	2.0	2.0	2.0	0.7	0.7	0.7	3.0	3.0	3.0	-	362	362
Madya Pradesh	1.1	1.1	1.1	1.2	1.2	1.2	1.0	1.0	1.0	-	-49	-49
Rajasthan	0.0	0.0	0.0	0.0	0.0	0.0	1.3	1.3	1.3	-	29	28
Uttar Pradesh	12.0	11.9	11.9	6.2	6.2	6.2	1.9	1.9	1.9	-	146	146
Uttarakhand	0.5	0.5	0.5	0.2	0.2	0.2	2.9	2.9	2.9	-	343	343
West Bengal	2.7	2.7	2.7	1.0	1.0	1.0	2.7	2.7	2.7	-	303	298
TOTAL	24.9	24.2	24.0	14.0	13.6	13.5						
	**production**			**area**			**yield**			**gross margin**		
**VARIABILITY**	**baseline**	**flexible**	**flex limited**	**baseline**	**flexible**	**flex limited**	**baseline**	**flexible**	**flex limited**	**baseline**	**flexible**	**flex limited**
Bihar	2.7	10.8	9.6	-	8.4	7.3	2.7	3.4	3.3	-	28.9	29.7
Haryana	5.3	5.3	5.3	-	0.0	0.0	5.3	5.3	5.3	-	8.9	8.9
Madya Pradesh	5.8	5.9	5.9	-	0.0	0.0	5.8	5.9	5.9	-	23.8	23.8
Rajasthan	5.3	5.1	4.8	-	0.0	0.0	5.3	5.1	4.8	-	49.6	47.4
Uttar Pradesh	6.8	7.1	7.1	-	0.2	0.2	6.8	7.1	7.1	-	19.0	19.1
Uttarakhand	4.2	4.2	4.2	-	0.0	0.0	4.2	4.2	4.2	-	7.3	7.3
West Bengal	3.2	3.2	3.4	-	0.0	2.8	3.2	3.2	3.1	-	5.7	5.6
**wheat**												
	**production**			**area**			**yield**			**gross margin**		
**AVERAGE**	**baseline**	**flexible**	**flex limited**	**baseline**	**flexible**	**flex limited**	**baseline**	**flexible**	**flex limited**	**baseline**	**flexible**	**flex limited**
Bihar	6.1	6.1	5.9	2.7	2.7	2.7	2.3	2.3	2.2	-	223	208
Haryana	5.2	5.1	5.1	1.2	1.2	1.2	4.3	4.3	4.3	-	627	627
Himachal Pradesh	0.0	0.0	0.0	0.0	0.0	0.0	1.7	1.7	1.7	-	99	99
Madya Pradesh	5.3	5.1	4.6	2.4	2.3	2.1	2.2	2.2	2.2	-	208	195
Rajasthan	4.1	3.8	2.8	1.6	1.5	1.1	2.6	2.6	2.5	-	284	273
Uttar Pradesh	24.9	24.8	24.3	10.0	10.0	9.8	2.5	2.5	2.5	-	264	261
Uttarakhand	1.2	1.2	1.2	0.3	0.3	0.3	4.4	4.4	4.4	-	661	661
West Bengal	0.3	0.3	0.2	0.1	0.1	0.1	2.1	2.0	1.9	-	173	138
TOTAL	47.1	46.4	44.2	18.3	18.0	17.3						
	**production**			**area**			**yield**			**gross margin**		
**VARIABILITY**	**baseline**	**flexible**	**flex limited**	**baseline**	**flexible**	**flex limited**	**baseline**	**flexible**	**flex limited**	**baseline**	**flexible**	**flex limited**
Bihar	4.1	4.1	3.8	-	0.1	1.4	4.1	4.1	4.4	-	8.7	9.5
Haryana	3.2	3.3	3.6	-	0.4	1.4	3.2	3.2	3.2	-	4.4	4.4
Himachal Pradesh	3.7	3.7	3.7	-	0.0	0.0	3.7	3.7	3.7	-	13.0	13.0
Madya Pradesh	4.1	4.5	7.3	-	2.9	5.9	4.1	4.1	3.7	-	9.0	8.4
Rajasthan	4.7	5.8	15.7	-	3.1	14.9	4.7	4.6	4.3	-	8.6	8.1
Uttar Pradesh	4.3	4.3	4.1	-	0.1	1.1	4.3	4.3	4.2	-	8.3	8.1
Uttarakhand	11.7	11.7	11.7	-	0.0	0.0	11.7	11.7	11.7	-	16.0	16.0
West Bengal	4.9	4.8	3.8	-	0.0	4.7	4.9	4.8	5.1	-	11.6	14.3

### Value of flexibility

**Fig 7:**
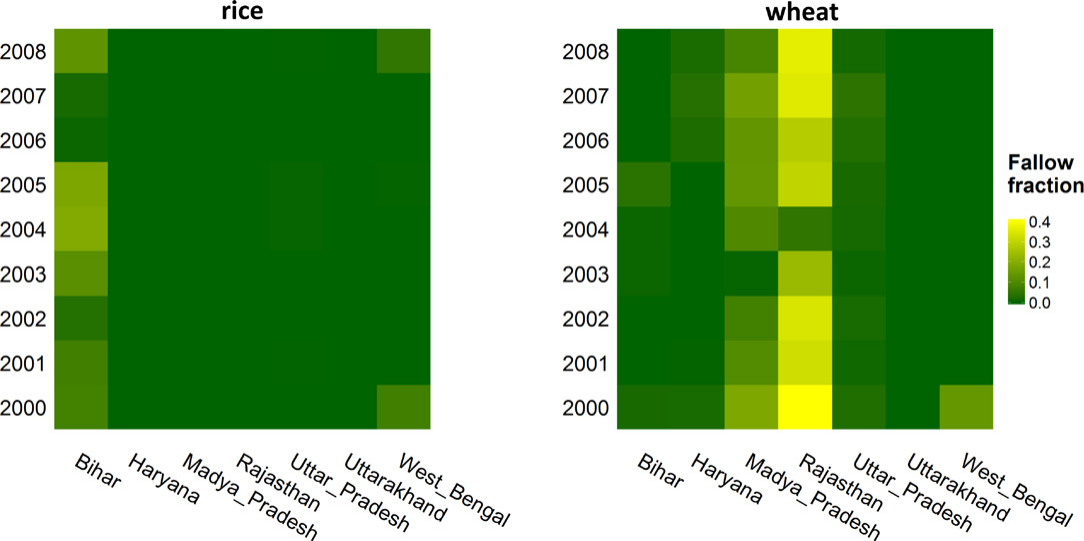
Annual fraction fallow land per state for rice (left) and wheat (right) in the “WW flexible-limited” variant.

The importance of being flexible in land use, i.e. being able to leave land fallow and reduce cropped area, differs for both rice and wheat and for the different states as Table 4 and Fig 7 show. Leaving land fallow is a strategy most relevant for wheat production, where especially in the state of Rajasthan the area left fallow is simulated to be high. In our model we find that in the drought year of 2002 the area cropped in Rajasthan was reduced by 34% compared to the maximum over the modelled period (2000–2009)–a percentage very similar to the reduction found in the statistics for the whole state of Rajasthan (minus 33%). For rice, only downstream Bihar and to a lesser extent West Bengal show comparatively small fluctuations in fallow area. In both states, rice is planted in a double crop rotation, so also during the *Rabi* cropping season in which we allowed the model to vary cropped area.

The ‘value of flexibility’ (VoF) becomes clear if we zoom in on wheat production in Rajasthan and we compare our final, *WW flexible-limited* variant to an alternative run with identical parameters settings, but without allowing the model to leave land fallow. Without this strategy of leaving land fallow during dry years, average crop productivity would go down by 20% to less than 2 ton/ha and economic yield per ha would be reduced by almost 40% to 168 USD/ha. Total gross margin from wheat production would be reduced by 12%.

**Fig 8:**
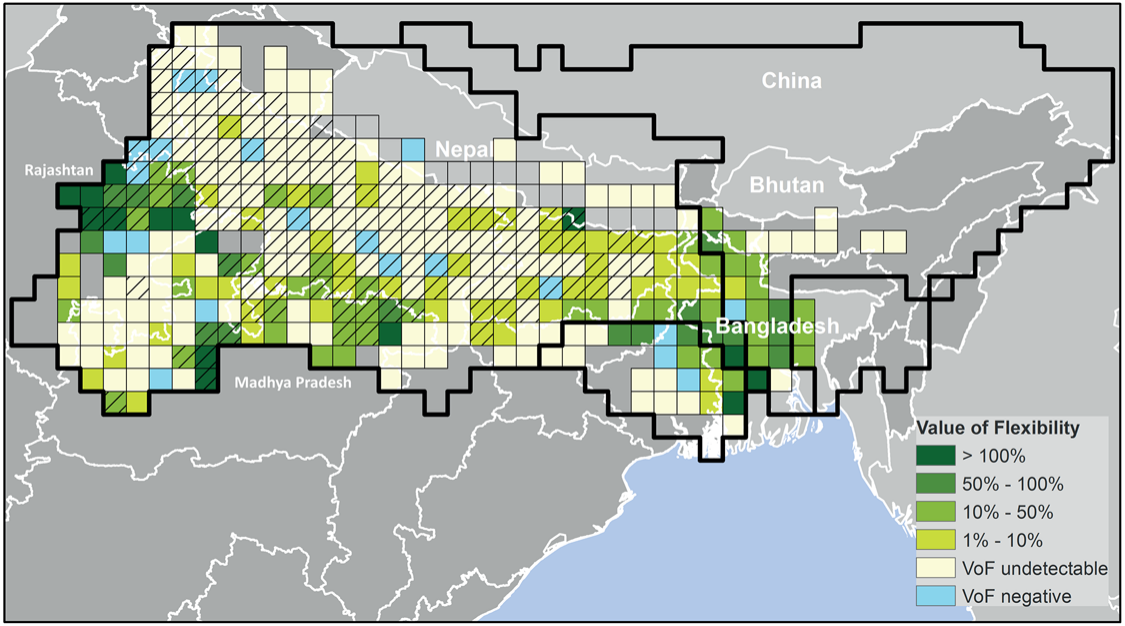
Value of flexibility for wheat production in the Ganges-Meghna-Brahmaputra basin (as percentage of gross margin). Shading indicates major wheat producing area (cells in which at least 20% of the area is cropped with wheat during the rabi season). In cells without color, along the Nepalese Himalayan foothills, wheat was not profitable in any year according to our model and therefore not cropped in the “WW flexible-limited” variant. In regions without cells, wheat is cropped on less than 2% of the area according to MIRCA2000.

This 12% can be considered the lower estimate of the VoF. In our validated model, we did not include the cost of family labor as we considered this was not a major decision factor in Indian agriculture over the past decades. However, with increased mobility and ongoing demand for labor in urban areas providing alternatives for on-farm family labor, and with rural employment and minimum wage schemes by the Indian government limiting the availability of hired labor in rural areas, costs of labor is rising [56] and likely to become a more prominent factor in farm-level decision making. If we would include the cost of labor, our expectation is that VoF will increase. To quantify this effect we increased cultivation costs by a third, the increase resembling the median costs of labor in major rice and wheat producing states, as calculated by the Indian Ministry of Agriculture for the year 2011/12 (http://eands.dacnet.nic.in, last visited 31-10-2014). In this scenario, up to two-thirds of the area is now left fallow in Rajasthan during years of water stress, while yield still remains at 2.6 ton/ha on those areas that are cropped and receive irrigation water. Compared to an alternative variant without flexibility, this leads to a difference in total gross margin from wheat in Rajasthan of 34%, a value that could be considered the VoF corrected for cost of labor. While important in drought prone states like Rajasthan, at basin level the VoF is limited, at 4% for wheat. Especially in the largest wheat producing state, Uttar Pradesh, wheat production remains fairly constant over the years in all scenarios (Fig 8).

Flexibility is beneficial for farmers’ gross margin, but it does come at a societal cost. When farmers are able to leave land fallow in order to maximize their returns, overall production decreases, potentially increasing the costs for consumers. In Rajasthan the decrease in production was 17%. At basin scale this effect on production was limited to a decrease of 4%.

For rice, with our current model setup and labour costs included, the VoF is close to zero as the largest share of production occurs during the monsoon season, for which we assumed land use is fixed. When we exclude labour costs, the VoF for rice is even slightly negative (-1%); if the model is not allowed to vary land use per season, more crops (e.g wheat, sugarcane) are irrigated upstream against high costs and return flows, also from groundwater, become available for rice cropping downstream. This slightly benefits rice production during the dry season especially in downstream Bihar. If farmers would have the option to choose, they would, however, still largely avoid rice production during dry years; for rice production in the *Rabi* season alone the VoF is 21%, indicating that in this season it can be a relevant coping strategy.

## Discussion

Simulation of agricultural production was improved by including seasonal decision making on cropped area in the hydro-economic model WaterWise. With the improved model we analyzed the impact of rainfall on the production of rice and wheat in the Indian part of the Ganges basin. The value of flexibility in cropped area was quantified for scenarios with and without costs of labor. While being high for wheat production in a drought prone state like Rajasthan, the value of flexibility was found to be limited for the Ganges basin as a whole, indicating that water resources are overall still largely sufficient, but unequally distributed.

We focused solely on the relationship between monsoon total rainfall and crop production variability, separating natural causes of inter-annual variability from socio-economic ones. The observed variability in production, and the fraction influenced by rainfall, can be regarded as the benchmark for the hydro-economic model. Inevitably, there are shortcomings in our approach, which make that simulated variability deviates from observed, such as:

1. It is not just total rainfall over the season but also its distribution that is critical for the replenishment of shallow reservoirs. It would be interesting to compare the Rabi cropped area decision and final yield and production between seasons of similar total rainfall, but a different distribution over the season;
2. We only considered decision making on cropped area for the second cropping season, *Rabi*, which starts after the monsoon. In order to better match observed variability in rice production, flexibility in planting during the *Kharif* season should be included. Climatic factors that trigger planting decisions during the *Kharif* season are, however, less straightforward. At the time of planting it is quite uncertain how the monsoon will unfold, so monsoon rainfall totals cannot be used as a decision-determining variable in a model. A more detailed assessment of the impact of late monsoon onset on cropping decisions or the use of seasonal forecasts would be required;
3. We focused on annual anomalies in production and rainfall. A drought, however, can have a prolonged effect, as the statistics on Rajasthan suggest. The area cropped with rice is suppressed for several years after the 2002 drought. Farmers might become more risk averse, or have less room for investments after an adverse weather year. Our model does not consider such inter-annual relationships. An option would be to expand the model with a farm-level budget, dependent on each year’s gross margin, from which land and water use investments in the next year have to be paid;
4. Our model inevitably oversimplifies a complex reality in which people might continue to grow crops based on cultural preferences, issues of food security or absence of alternatives (to mention a few of possible factors), rather than applying an economic logic solely based on cost and benefits and the availability of water. Farmers with less entitlement or access to water might refrain from planting a second crop when rainfall is not far from the mean, while others irrigate too much. While this would show up in the statistical data, the model does not consider this aspect;
5. In addition, we used fixed prices for agricultural output, independent of total production. In theory, prices will rise during years of shortage, favoring planting rather than leaving land fallow. Farmers with access to additional irrigation or other inputs might anticipate such higher prices and plant more, rather than less in adverse climate years. Locally, this would reduce flexibility. That said, in India prices of major food commodities like rice and wheat are controlled by the government, so this incentive is expected to be less prominent in our case study area.
6. Finally, we did not allow our model to switch to other crops in this application, though this is a relevant strategy in dealing with rainfall variability. Farmers sometimes shift from food to fodder crops, for example, during drought to increase their income from dairy production. Incorporating such shifts, including their costs, would enable evaluating a combination of strategies, including both diversification and flexibility.

Despite these shortcomings, our improved model was capable of simulating existing variability at different spatial aggregation levels, especially for wheat. The model mimics the strategy of farmers to concentrate cultivation and irrigation on a smaller area in years of shortage, especially during the second growing season. In a sense this is a second-best strategy: farmers prefer a constant maximum use of land. But when water is not sufficiently available at reasonable costs, avoiding loss of investments becomes the main strategy. With rainfall variability expected to increase due to climate change and costs of groundwater irrigation likely to rise due to falling groundwater levels and/or a reduction in subsidies on diesel or electricity, a higher variability in production can be expected.

Improved understanding of seasonal variability in food production is important for policy makers and planners dealing with food security, both regionally and globally. While India is largely food self-sufficient now, a major question is to what extent variability will affect it in the future, when a growing population will put more pressure on limited land and water resources. Understanding variability is thereby not only of relevance for coping with shortages, but also for efficiently managing surpluses; both the amplitude of fluctuations in production and the frequency of extremes influence the stocks that need to be kept and the volume that can be exported.

Flexibility in land use should be seen as a vital coping strategy for dealing with water shortages due to rainfall variability. Coping with current variability is often considered as a first step towards coping with future climate change [57,58]. An analysis of how increased variability in rainfall might lead to permanent changes in cropping pattern, or a permanent reduction in cropped area, would remain a relevant next step to explore.

## Conclusions

Seasonal adjustment in cropped area can explain almost 50% of variability in wheat production and 40% variability in rice production in the Indian part of the Ganges basin. This makes these adjustments almost as important as variability in yield. The distinction matters economically; while changes in cropped area represent a coping strategy for adverse conditions, a reduction in yield is merely a response of the crop. In both cases production and income are reduced. But when a farmer can decide not to crop, costs can be avoided as well.

Our improved hydro-economic model, with the capacity to seasonally adjust cropped area and irrigation application, is capable of reproducing observed rainfall-induced variability in wheat production at district, state and basin level, but is at the lower end of observed variability for rice. Wheat production is most influenced by limitations to the availability of groundwater. Rice production reacts mainly to increased costs of cultivation.

The value of flexibility, i.e. the benefit of being able to adjust cropped area, was estimated for wheat at 34% (increase in gross margin) in the drought prone state of Rajasthan and at 4% for the basin as a whole. For rice, the area cropped was largely stable in our model, and variability in rice production was at the lower end of the expected observed variability. A better understanding of the impact of seasonal forecasts, monsoon onset and break-monsoon periods during transplanting time, a critical moment in crop management, could improve our assessment of the variability and the value of flexibility in rice production and other crops grown during the monsoon.

## Supporting Information

S1 File Short summary of WaterWise model equations. (DOCX)

S2 File Changes in variability of crop production, yield and area in India. (DOCX)

S3 File Correlation between inter-annual rainfall anomalies and crop production anomalies. (DOCX)

## References

[1] Joseph P, Sabin T (2008) An ocean–atmosphere interaction mechanism for the active break cycle of the Asian summer monsoon. Climate dynamics 30: 553–566.

[2] Annamalai H, Slingo J (2001) Active/break cycles: diagnosis of the intraseasonal variability of the Asian summer monsoon. Climate Dynamics 18: 85–102.

[3] Singh D, Tsiang M, Rajaratnam B, Diffenbaugh NS (2014) Observed changes in extreme wet and dry spells during the South Asian summer monsoon season. Nature Climate Change 4: 456–461.

[4] Meehl GA (1997) The South Asian Monsoon and the Tropospheric Biennial Oscillation. Journal of Climate 10: 1921–1943.

[5] Krishnamurthy V, Shukla J (2000) Intraseasonal and Interannual Variability of Rainfall over India. Journal of Climate 13: 4366–4377.

[6] Abish B, Joseph P, Johannessen OM (2013) Weakening trend of the tropical easterly jet stream of the boreal summer monsoon season 1950–2009. Journal of Climate 26: 9408–9414.

[7] Krishnamurthy V, Goswami BN (2000) Indian Monsoon–ENSO Relationship on Interdecadal Timescale. Journal of Climate 13: 579–595.

[8] Joseph P, Bindu G, Nair A (2013) Variability of summer monsoon rainfall in India on in-ter-annual and decadal time scales. Atmos Oceanic Sci Lett 6: 398–403.

[9] Mathison C, Wiltshire A, Dimri A, Falloon P, Jacob D, Kumar P, et al (2013) Regional projections of North Indian climate for adaptation studies. Science of The Total Environment 468: S4–S17. doi: 10.1016/j.scitotenv.2012.04.066 22633462 10.1016/j.scitotenv.2012.04.066

[10] Turner AG, Annamalai H (2012) Climate change and the South Asian summer monsoon. Nature Climate Change 2: 587–595.

[11] Sharmila S, Joseph S, Sahai AK, Abhilash S, Chattopadhyay R (2015) Future projection of Indian summer monsoon variability under climate change scenario: An assessment from CMIP5 climate models. Global and Planetary Change 124: 62–78.

[12] Kumar P, Wiltshire A, Mathison C, Asharaf S, Ahrens B, Lucas-Picher P, et al (2013) Downscaled climate change projections with uncertainty assessment over India using a high resolution multi-model approach. Science of The Total Environment 468–469, Supplement: S18–S30. 10.1016/j.scitotenv.2013.01.051 23541400

[13] Krishna Kumar K, Rupa Kumar K, Ashrit RG, Deshpande NR, Hansen JW (2004) Climate impacts on Indian agriculture. International Journal of Climatology 24: 1375–1393.

[14] Kumar R, Singh RD, Sharma KD (2005) Water resources of India. Current science 89: 794–811.

[15] Parthasarathy B, Munot AA, Kothawale DR (1988) Regression model for estimation of indian foodgrain production from summer monsoon rainfall. Agricultural and Forest Meteorology 42: 167–182.

[16] Revadekar JV, Preethi B (2012) Statistical analysis of the relationship between summer monsoon precipitation extremes and foodgrain yield over India. International Journal of Climatology 32: 419–429.

[17] IMD (2015) http://www.imd.gov.in/doc/wxfaq.pdf. Pune: Indian Meteorological Department.

[18] Siderius C, Hellegers PJGJ, Mishra A, van Ierland EC, Kabat P (2013) Sensitivity of the agroecosystem in the Ganges basin to inter-annual rainfall variability and associated changes in land use. International Journal of Climatology: n/a-n/a.

[19] GoI (2012) Agricultural Statistics at a glance 2012. New Delhi: Government of India, Ministry of Agriculture.

[20] Chambers R (1988) Managing canal irrigation: Practical analysis from South Asia: Cambridge University Press.

[21] Meinzen-Dick R, Raju KV, Gulati A (2002) What Affects Organization and Collective Action for Managing Resources? Evidence from Canal Irrigation Systems in India. World Development 30: 649–666.

[22] Siderius C, Boonstra H, Munaswamy V, Ramana C, Kabat P, van Ierland E, et al (2015) Climate-smart tank irrigation: A multi-year analysis of improved conjunctive water use under high rainfall variability. Agricultural Water Management 148: 52–62.

[23] Venot J, Jella K, Bharati L, George B, Biggs T, Rao P, et al (2010) Farmers’ Adaptation and Regional Land-Use Changes in Irrigation Systems under Fluctuating Water Supply, South India. Journal of Irrigation and Drainage Engineering 136: 595–609.

[24] Kelkar U, Narula KK, Sharma VP, Chandna U (2008) Vulnerability and adaptation to climate variability and water stress in Uttarakhand State, India. Global Environmental Change 18: 564–574.

[25] Arnold JG, Fohrer N (2005) SWAT2000: current capabilities and research opportunities in applied watershed modelling. Hydrological Processes 19: 563–572.

[26] Best MJ, Pryor M, Clark DB, Rooney GG, Essery RLH, Ménard CB, et al (2011) The Joint UK Land Environment Simulator (JULES), Model description–Part 1: Energy and water fluxes. GeoScientific Model Development 4: 595–640.

[27] Gerten D, Schaphoff S, Haberlandt U, Lucht W, Sitch S (2004) Terrestrial vegetation and water balance—Hydrological evaluation of a dynamic global vegetation model. Journal of Hydrology 286: 249–270.

[28] Liang X, Lettenmaier DP, Wood EF, Burges SJ (1994) A Simple hydrologically Based Model of Land Surface Water and Energy Fluxes for general circulation models. Journal of Geophysical Research 99: 415–428.

[29] Kummu M, Gerten D, Heinke J, Konzmann M, Varis O (2014) Climate-driven interannual variability of water scarcity in food production potential: a global analysis. Hydrology and Earth System Sciences 18: 447–461.

[30] Wada Y, van Beek LPH, van Kempen CM, Reckman JWTM, Vasak S, Bierkens MFP (2010) Global depletion of groundwater resources. Geophysical Research Letters 37: L20402.

[31] Grömping U (2006) Relative importance for linear regression in R: the package relaimpo. Journal of statistical software 17: 1–27.

[32] Chevan A, Sutherland M (1991) Hierarchical partitioning. The American Statistician 45: 90–96.

[33] Asseng S, Ewert F, Rosenzweig C, Jones JW, Hatfield JL, Ruane AC, et al. (2013) Uncertainty in simulating wheat yields under climate change. 3: 827–832.

[34] Biemans H, Siderius C, Mishra A, Ahmad B (2015) Crop-specific seasonal estimates of irrigation water demand in South Asia. Hydrology and Earth System Sciences Discussions.

[35] Cai X (2008) Implementation of holistic water resources-economic optimization models for river basin management–reflective experiences. Environmental Modelling & Software 23: 2–18.

[36] Cai X, McKinney DC, Lasdon LS (2003) Integrated hydrologic-agronomic-economic model for river basin management. Journal of water resources planning and management 129: 4–17.

[37] Yang Y-CE, Brown CM, Yu WH, Savitsky A (2013) An introduction to the IBMR, a hydro-economic model for climate change impact assessment in Pakistan’s Indus River basin. Water International 38: 632–650.

[38] Portmann FT, Siebert S, Döll P (2010) MIRCA2000—Global monthly irrigated and rainfed crop areas around the year 2000: A new high‐resolution data set for agricultural and hydrological modeling. Global Biogeochemical Cycles 24.

[39] FAO (2015) FAO AQUASTAT, www.fao.org/nr/water/aquastat/main/index.stm. Rome.

[40] Siderius C, Hellegers P, Mishra A, van Ierland E, Kabat P (2014) Sensitivity of the agroecosystem in the Ganges basin to inter‐annual rainfall variability and associated changes in land use. International Journal of Climatology 34: 3066–3077.

[41] Whittington D, Wu X, Sadoff S (2005) Water resources management in the Nile basin: the economic value of cooperation. Water policy 7: 227–252.

[42] Wu X, Jeuland M, Sadoff C, Whittington D (2013) Interdependence in water resource development in the Ganges: an economic analysis. Water Policy 15: 13–09.

[43] van Walsum P, Helming J, Stuyt L, Schouwenberg E, Groenendijk P (2008) Spatial planning for lowland stream basins using a bioeconomic model. Environmental Modelling & Software 23: 569–578.

[44] FICO (2014) FICO Xpress optimization suite. http://www.fico.com/en/products/fico-xpress-optimization-suite/. pp. http://www.fico.com/en/products/fico-xpress-optimization-suite/.

[45] Rost S, Gerten D, Bondeau A, Lucht W, Rohwer J, Schaphoff S (2008) Agricultural green and blue water consumption and its influence on the global water system. Water Resources Research 44: W09405.

[46] Falkenmark M, Rockström J, Karlberg L (2009) Present and future water requirements for feeding humanity. Food Security 1: 59–69.

[47] Biemans H (2012) Water constraints on future food production Wageningen: Wageningen UR.

[48] Sitch S, Smith B, Prentice IC, Arneth A, Bondeau A, Cramer W, et al (2003) Evaluation of ecosystem dynamics, plant geography and terrestrial carbon cycling in the LPJ dynamic global vegetation model. Global Change Biology 9: 161–185.

[49] Bondeau A, Smith PC, Zaehle S, Schaphoff S, Lucht W, Cramer W, et al (2007) Modelling the role of agriculture for the 20th century global terrestrial carbon balance. Global Change Biology 13: 679–706.

[50] Weedon GP, Balsamo G, Bellouin N, Gomes S, Best MJ, Viterbo P (2014) The WFDEI meteorological forcing data set: WATCH Forcing Data methodology applied to ERA‐Interim reanalysis data. Water Resources Research 50: 7505–7514.

[51] Yadav R, Rao S (2001) Atlas of cropping systems in India: Project Directorate for Cropping Systems Research (ICAR).

[52] GoI (2008) Manual on cost of cultivation surveys New Delhi: Government of India, Central statistical organization.

[53] Siebert S, Döll P, Feick S, Hoogeveen J, Frenken K (2007) Global map of irrigation areas version 4.0.1 Johann Wolfgang Goethe University, Frankfurt am Main, Germany/Food and Agriculture Organization of the United Nations, Rome, Italy.

[54] Shah T (2010) Taming the anarchy: Groundwater governance in South Asia: Routledge.

[55] Kannan E, Sundaram S (2011) Analysis of trends in India’s agricultural growth. The Institute for Social and Economic Change, Bangalore, India, Working paper 276.

[56] GoI (2013) State of Indian Agriculture 2012–13. New Delhi: Government of India, Ministry of Agriculture.

[57] Glantz MH (1992) Global warming and environmental change in sub-Saharan Africa. Global Environmental Change 2: 183–204.

[58] Kabat P, Schulze RE, Hellmuth ME, Veraart JA (2002) Coping with impacts of climate variability and climate change in water management: a scoping paper. Wageningen.

