# Supplementary material for: Flexible Strategies for Coping with Rainfall Variability: Seasonal Adjustments in Cropped Area in the Ganges Basin
Source: PLoS One. 2016 Mar 2;11(3):e0149397. doi: 10.1371/journal.pone.0149397 (PMC4774993; doi:10.1371/journal.pone.0149397)
Supplement: S1 File — (DOCX) [file pone.0149397.s001.docx]

**SUPPLEMENTARY INFORMATION 1**

**Short summary of WaterWise model equations**

***Introduction***

The WaterWise model has the specific ability to suggest investments that make best use of the available land and water resources. It solves the problem of economic scarcity, with the implementation of local investments having consequences for the physical possibility of investments elsewhere. Like most hydro-economic models, WW describes the hydrologic and crop growth processes in considerable detail, whereas the economic optimization algorithm is relatively simple.

WaterWise is a hybrid-holistic model: separate water-crop modules are run in an offline mode as part of the preprocessing. The results are imported into the optimization model through continuous decision variables on the interval [0,1] that represent the area fraction for which the option is actually applied: attached to these variables are all the (time dependent) water balance variables and crop production variables of a certain crop management option. The attached variables can have any kind of nonlinear interaction with each other, since this does not have to be formally represented in the hybrid holistic model. In this manner the (extreme) nonlinearities between water and crop production in the column model are modelled with linear variables in the hybrid holistic model.

The Waterwise model code is formulated within a Mixed Integer Linear Programming framework (MILP). The model equations have been implemented in Xpress-Mosel (FICO, 2014)[[1]](#footnote-1). The MILP technique is for instance used in representing discrete options like the building of a reservoir. There are many examples in literature of this usage, e.g. Gillig et al. (2001)[[2]](#footnote-2). Less common is the use of MILP for representing nonlinear hydrologic relationships and thresholds in the economic evaluation. In comparison to nonlinear techniques involving a gradient search, mixed integer linear programming has the advantage that when the optimum is found one can be sure it is the global optimum, without any further analysis required.

***Land use model***

The land use and management options are modelled with decision variables on the interval [0,1]. The constraint for the choice between land use options reads as:

${\sum_{u} XU_{z,u}= 1}$ *{22}*

where *XU_z,u_* is the decision variable for the fraction of land use option *u* in hydrotope *z* (-).The model contains possibilities for constraining the total fraction of the hydrotope area that can be converted, limiting it to e.g. 20%. It is also possible to set a constraint on the fraction of the area that can be converted to a certain land-use type. In addition, the land use options can also be clustered into groups, e.g. of ‘cereals’ and ‘other’ crops. The model is then forced to keep the total area of the group the same, within each hydrotope or within a certain region.

For estimating the costs of transitions, the land use changes that the model is generating are compared with the current situation by:

${\sum_{u2} X{U2XU}_{z,u2,u}- \sum_{u2} X{U2XU}_{z,u,u2}= XU_{z,u}}- {{aluref}_{z,u}}/{A_{z}}$ *{23}*

where *XU2XU_z,u2,u_* is the decision variable for conversion of land-use type *u2* to *u* in hydrotope z, *aluref_z,u_* is the area of land use option *u* in a hydrotope in the current situation (ha), and *A_z_* the area of hydrotope *z* (ha). By attaching cost coefficients to the changes, the model is encouraged to select values of variables involving minimal changes; this resolves the problem of indeterminacy due to the presence of more variables than equations.

***Water model***

In the water model the decision variable decision variable *X*_z,u,m,y,s_ represents the use of a management option *m* of land use type *u* in hydrotope *z*, in season *s* of year *y*. The water model connects to the land use model by setting the sum of the used management options equal to the land use option

$$\sum_{m} X_{\text{z,u,m,y,s}} = XU_{\text{z,u}}$$

The use of a land and water management option can involve costs, which are determined by the maximum value of *X* that is chosen for the modelling period. The runoff and drainage of land use (both agriculture and non-agriculture) are summated for the node that the flow goes to:

$$QRK_{\text{k,t}}=\sum_{\text{z|koutz(z)}=\text{k}} \sum_{\text{u,m}} X_{\text{z,u,m,y(t),s(t)}}\cdot A_{\text{z}} \cdot(qdrn_{\text{z,u,m,t}} +qroff_{\text{z,u,m,t}})$$

where *QRK*_k,t_  is the sum of runoff and drainage connected to node *k* (m^3^ s^-1^), *qdrn*_z,u,m,t_ and *qroff* _z,u,m,t_ are drainage and runoff (m^3^ s^-1^ m^--2^). The latter parameters are determined with offline running of the water module in a pre-processing stage.

For modelling water demand the model formulation is more complex, because demand realization depends on water supply decisions that can be flexible, from time step to time step, with water coming from varying sources at specific costs per unit, and with specific constraints (physical or policy driven). The demand realization can come from a local source (groundwater, local surface water) and/or a regional source (the main river). For supply from local groundwater there is not a connection to the network of water bodies. The supply is based on simulations preformed offline in the pre-processing phase for the vertical groundwater-soil-crop column, assuming that no groundwater mining is allowed. Whether or not the option is used depends on the decision variable *X*_z,u,m,y,s_ . Supply from regional groundwater and from surface water can be from a node or from an arc of the water network. The chain of equations for water demand satisfaction starts with:

$$\sum_{\text{v}} (QSKV_{\text{v,z,u,t}}+QSJV_{\text{v,z,u,t}}) =\sum_{\text{m}} X_{\text{z,u,m,y(t),s(t)}} \cdot Ac_{\text{z}} \cdot qdem_{\text{z,u,m,t}}$$

where *QSKV*_v,z,u,t_ is the irrigation supply from a node that has been labelled as type of source *v (*groundwater, local surface water, river water*)* (m^3^ s^-1^), *QSJV*_v,z,u,t_ is the irrigation supply from an arc that has been labelled as source *v* (m^3^ s^-1^), *Ac_z_* is cropped area (in ha) and *qdem*_z,u,m,t_ is the irrigation demand determined with running the offline water module (m^3^ s-^1^m^-2^). The above equation can lead to model infeasibility if there is not enough water. To avoid this, the model application should always include a ‘rainfed’ option that has no irrigation demand. The model can then selectively use this option in a season with a shortage of water, and in the rest of the seasons use the option with the irrigation enabled. Via the yield coefficients in the objective function the loss of productivity is taken into account.

The irrigation supply from nodes and arcs connect to the water network with:

$QSK_{\text{k,t}} =\sum_{\text{v,u,z | kinz}_{\text{v,z}}=\text{k}} QSKV_{\text{v,z,u,t}}$ ; $QSJ_{\text{j,t}} =\sum_{\text{v,u,z | jinz}_{\text{v,z}}=\text{j}} QSJV_{\text{v,z,u,t}}$

where *kinz(v,z)* links a hydrotope to a water body node, and *jinz(v,z)* links a hydrotope to a water body arc. The supply of water can involve costs. The required supply capacity is determined by the maximum supply rate in the modelling period. This can be limited due to physical or cost considerations.

The nodes only act as connection hubs, without any spatial dimension or storage:

$$\sum_{\text{j | koutj}_{\text{j}}=\text{k}} QFOUTJ_{\text{j,t}}+QRK_{\text{k,t}}=\sum_{\text{j | kinj}_{\text{j}}=\text{k}} QFINJ_{\text{j,t}}+ QSK_{\text{k,t}}$$

where *QFOUTJ*_j,t_ is the outflow of arc *j* (m^3^ s^-1^), and *QFINJ*_j,t_ is the inflow of arc *j* (m^3^ s^-1^).

To model the actual flow through an arc of the network we used the unit hydrograph method (UH). This method provides a means to introduce extra translation time and extra flood wave dispersion. Losses can be modelled schematically by letting the blocks of the UH add up to less than the unit.

***Reservoir model***

For reservoirs we used a variable storage routing method, which includes an area dependent recharge/loss term attached to the arcs:

$$S_{\text{j,t}}=S_{\text{j,t-1}}+[QFINJ_{\text{j,t}}-QFOUTJ_{\text{j,t}} - QSJ_{\text{j,t}} +A_{\text{j,t}}\cdot recha_{\text{j,t}}]\Delta t$$

where *S*_j,t_ is the storage in an arc *j* (m^3^), *A*_j,t_ is the water area (m^2^), *recha*_j,t_ is the recharge/loss term (m^3^ s^-1^ m^-2^), *Δt* is the length of time interval (s). In order to avoid the non-sustainable use of a reservoir, the model sets the storage at the end of the simulation run equal to that at the beginning, with the model itself determining that storage as part of the optimization. If the latter feature is not desired, a minimum and/or maximum end storage can be specified.

Piece-wise linear functions are used for modelling the relationships between water level, storage and gate outflow capacity of the arc/reservoir itself. The implementation is done with a ‘special ordered set of type 2’, a so-called SOS2-set of ordered decision variables in the form of weights (Fico, 2014). Such a set ensures that the model is forced to follow a nonlinear table, without ‘cutting corners’. The equations that make parallel use of the SOS2-weight variables are given by:

$H_{\text{j,t}}=\sum_{\text{p}\in\text{SOS2}} hsos_{\text{j,p}}\cdot WT_{\text{j,t,p}}$ ; $A_{\text{j,t}}=\sum_{\text{p}\in\text{SOS2}} asos_{\text{j,p}}\cdot WT_{\text{j,t,p}}$; $S_{\text{j,t}}=\sum_{\text{p}\in\text{SOS2}} ssos_{\text{j,p}}\cdot WT_{\text{j,t,p}}$

$QFOUTJ_{\text{j,t}} =\sum_{\text{p}\in\text{SOS2}} qsos_{\text{j,p}}\cdot WT_{\text{j,t,p}}$ ; $\sum_{\text{p}\in\text{SOS2}} WT_{\text{j,t,p}} = 1$

where *WT*_j,t,p_ is a weight variable of the piece-wise linear function, table position *p* (-), *H*_j,t_ is the water level (in m) in arc *j* at time *t*, *hsos*_j,p_ is a water level point of piece-wise linear function (m), *asos*_j,p_ is a surface water area point of piece-wise linear function (m^2^), *ssos*_j,p_ is a surface water storage point of piece-wise linear function (m^3^), and *qsos*_j,p_ is discharge of piece-wise linear function 1 (m^3^ s^-1^).

For simulation of the spillway discharge the table can include an extra discharge term, or the network can include the spillway as a bypass. Lateral losses to groundwater can be modelled with an arc-arc connection. The use of integer variables is computationally demanding and therefore reserved for large reservoirs in the main river system that have a large evapotranspiration that is sensitive for the water area. Apart from restricted use of the option, the used time step is substantially longer than used for the rest of the system description.

For modelling local storage in surface water and groundwater, the so-called V-reservoirs, the used tables have just two entries: one starting at zero, the other for the maximum storage situation. Since the table function of these reservoirs only have two points, there is no need for using the computationally demanding SOS2-set, which makes the implementation of V-reservoirs straightforward LP.

***Flow boundary conditions***

Outflows of an arc can be set to a maximum, which is especially relevant for canal offtakes from the main river system and for limiting the infiltration capacity to a groundwater body. The defined water network can include arcs that do not actually exist yet. In that case an investment will be required. To describe this, the model has binary variables for activating the arc. If there are multiple parallel options for a new connection, then the user can specify that only one of them can be chosen. Environmental flows can be specified as a minimum flow for each time step, or as a long term average over the full period of the simulation.

***Crop production model***

In the integrated code of the optimization model, crop productivity is represented by coefficients that have been determined by running the crop production model in an offline mode (in this case LPJmL). Crop productivity is linked to the decision variable *X*_z,u,m,y,s_.

***Hydropower model***

For modelling hydropower there are two options:

- a linear relationship between flow (*QFOUTJ*) and generated power;
- a nonlinear relationship between head (*H*), flow (*QFOUTJ*) and generated power.

The nonlinear option is implemented with a so-called SOS1 set (FICO, 2014), that makes use of the water level modelled with the SOS2-set of the variable storage routing method. The hydropower model was not used in the Ganges-Meghna-Brahmaputra application.

***Economic model***

WW optimizes the total Gross Margin (total yield-over-cost), choosing the optimal combination of land use and water management options, given available water resources:

with *[2]*

$$Y_{LU} = \sum_{z,u,y,s} ({Prod}_{z,u,y,s} * P_{y,u} - C_{LU,u} * {Ac}_{z,u,y,s})$$

$$C_{LWM} = \sum_{z,u,y,s} (C_{IRRIz,u}* {Ac}_{z,u,y,s})$$

where *Y_TOT_* represents total gross margin (Indian Rupee (e.g. in Indian Rupees [Rp]) /yr), *Y_LU_*  the profit from land use (Rp/yr) based on production (*Prod*, in ton) times price of product (*P*, Rp/ton) minus non-water costs *(C_LU_*, Rp/ha) times the cropped area *(Ac*, in ha), in season *s* of year *y* per land use *u* in hydrotope *z. C_LWM_* are the costs of local water-management measures for supporting land use, i.e., the variable costs of local irrigation measures (in Rp/yr), depending on the amount of irrigation water used for each hydrotope *z* an land use and water management option *u*. *Y_HP_* are the gross margin of hydropower (Rp/yr), based on flow through the hydropower arc (QSOUTJ) times the hydropower-station specific yield (in Rp /m^3^). *C_RWM_* the costs of regional water management (i.e., maintenance costs for large canals and the costs of flow-through connections that involve pumping to support the river, canal, and reservoir system [Rp/yr]). Y_HP_ and C_RWM_ were not used in the Ganges-Meghna-Brahmaputra application.

In addition, investment costs can be inserted for modifications to the land use and water management system:

*I = I_LU_ + I_LWM_ + I_HP_ + I_RWM_*

where *I* is total investment (in e.g Rp ), *I_LU_* is investments in transitions of land use (Rp), *I_LWM_* is investments in improving local water management (Rp), *I_HP_* is investments in hydropower (Rp) and *I_RWM_* is investments in regional water management (Rp ). These investment costs can be annualized and added to the yield term or kept separate and given an upper bound. In the Ganges-Meghna-Brahmaputra application, no investment costs were used as we assessed present-day variability and the overall cropping pattern and water management structure was kept constant.

1. FICO, 2014. FICO Xpress optimization suite. http://www.fico.com/en/products/fico-xpress-optimization-suite/, pp. http://www.fico.com/en/products/fico-xpress-optimization-suite/. [↑](#footnote-ref-1)
2. Gillig, D., B. A. McCarl and F. Boadu 2001. An economic, hydrologic, and environmental assessment of water management alternative plans for the south central Texas region, Journal of Agricultural Applied Economics 33:59–78. [↑](#footnote-ref-2)
