# Supplementary material for: Flexible Strategies for Coping with Rainfall Variability: Seasonal Adjustments in Cropped Area in the Ganges Basin
Source: PLoS One. 2016 Mar 2;11(3):e0149397. doi: 10.1371/journal.pone.0149397 (PMC4774993; doi:10.1371/journal.pone.0149397)
Supplement: S2 File — (DOCX) [file pone.0149397.s002.docx]

**SUPPLEMENTARY INFORMATION 2: Changes in variability of crop production, yield and area in India**

Overall, at all-India level, fluctuations in crop production have increased in absolute terms, but decreased in relative terms, as a result of large increases in area, yield and production over the past decades (S2 table A). We split the data in three parts: 1950-1965, representing the pre-Green revolution period, 1965-1990 the era of cropland expansion and increases in yield and production and 1991 – 2013 as the liberalization period in which India opened its markets and liberalized its economy. For both rice and wheat absolute variability in production has increased over the periods considered. But, as total production has increased faster, relative variability in production has decreased from 11.4% (for rice) and 15.6% (for wheat) in the first period cropped to 6.4% (for rice) and 6.3% (for wheat) in the most recent period. Yield variability shows a similar pattern. Only the change in variability in area differs between rice and wheat; fluctuations in cropped area of rice have become larger while those in cropped area of wheat seem to have become smaller in both absolute and relative terms.

**S2 Table A** changes in absolute and relative cropped area, yield and production variability for rice and wheat in India (expressed as standard deviation and relative standard deviation)

* P < 0.1 (double-sided F-test)

** means P< 0.05 (double-sided F-test)
