# Supplementary material for: Flexible Strategies for Coping with Rainfall Variability: Seasonal Adjustments in Cropped Area in the Ganges Basin
Source: PLoS One. 2016 Mar 2;11(3):e0149397. doi: 10.1371/journal.pone.0149397 (PMC4774993; doi:10.1371/journal.pone.0149397)
Supplement: S3 File — (DOCX) [file pone.0149397.s003.docx]

**SUPPLEMENTARY INFORMATION 3: Correlation between inter-annual rainfall anomalies and crop production anomalies**

To get an indication how much of the observed variability in production is associated with rainfall, we estimated the correlation between rainfall anomalies (for the monsoon, i.e., the total of JJAS months , data taken from the 1 by 1 degree gridded data product from the Indian Meteorological Department (2)) and anomalies in de-trended annual production data. R-squared of such a correlation represents the explained variance, in this case by rainfall, divided by the total variance. From this it follows that Pearson’s r represents the explained standard deviation (σ_prod_). We multiplied the Pearson’s r value with observed σ_prod_ for the shorter time series for our model domain at different spatial aggregation levels (district, state, basin), to get an estimate of rainfall-induced variability at each level. This allowed us to compare results of the model with observations. We thereby assume that the influence of rainfall variability on rice and wheat production in the Ganges basin is similar to that for the whole of India, an assumption that seems reasonable as approximately 50% of rice and 70% of wheat production occurs in states within in the basin (1). We used a Fisher’s z’ transformation to calculate the upper and lower bound (90% confidence interval, n = 57) around the Pearson’s r value.

We found the correlation between monsoon rainfall and de-trended production anomalies to be of medium strength, with a Pearson’s r of 0.61 for rice and Pearson’s r of 0.50 for wheat for the whole of India over the period 1951 – 2007, indicating that 61% and 50% of standard deviation in production (σ_prod_) can be explained by rainfall variation. Applying a 90% confidence interval, using the Fisher’s z transformation, gave an indicative range for the influence of rainfall variations on rice σ_prod_ from 46% to74%. For wheat, the indicative range is from 31% to 65%, (as reflected by the uncertainty ranges, “*Observed rain-induced”,*  in figure 4 and 6 in the main text). In an extensive earlier study on the relation between climate and food production in India, Krishna Kumar *et al*.(3) found r = 0.77 for the correlation between rice production anomalies and rainfall anomalies and r= 0.47 for wheat. Mainly the difference in de-trending method – they took the relative difference between the value in one year compared to the previous year – explains the lower correlation for rice in our analysis*.*

1. GoI. Agricultural Statistics at a glance 2012. New Delhi: Government of India, Ministry of Agriculture, 2012.

2. Rajeevan M, Bhate J, Kale J, Lal B. High resolution daily gridded rainfall data for the Indian region: Analysis of break and active monsoon spells. Current Science. 2006;91(3):296-306.

3. Krishna Kumar K, Rupa Kumar K, Ashrit RG, Deshpande NR, Hansen JW. Climate impacts on Indian agriculture. Int J Climatol. 2004;24(11):1375-93.
